# Terra firma-forme dermatosis: an underdiagnosed condition^☆^^☆☆^

**DOI:** 10.1016/j.abd.2019.07.013

**Published:** 2020-03-20

**Authors:** Bruna Anjos Badaró, Lucia Martins Diniz, Paulo Sergio Emerich Nogueira

**Affiliations:** Service of Dermatology, Hospital Universitário Cassiano Antônio Moraes, Universidade Federal do Espírito Santo, Vitória, ES, Brazil

Dear Editor,

A 12-year-old girl, who had no co-morbidities, sought dermatological care due to the emergence of brownish spots that looked like “dirt”, which were spread through the body and had progressively evolved in the previous two years. This condition caused great social impact to the patient, given that she suffered from discrimination at school. She had used ketoconazole cream as prescribed by a physician of the Basic Health Unit; however, there was no clinical response. The patient noticed that the lesions became discreetly clearer after intense rubbing with gauze, but without improvement after washing with soap and water.

The dermatological examination indicated that the patient had hyperkeratotic brownish plaques with a dotted pattern, affecting the neck, back, abdomen, and, in a less evident manner, the lower limbs (Figure 1, Figure 2). After suspecting the presence of terra firma-forme, the lesions were rubbed with gauze embedded in 70% alcohol and the plaques were removed (Fig. 3).

**Figure 1:**
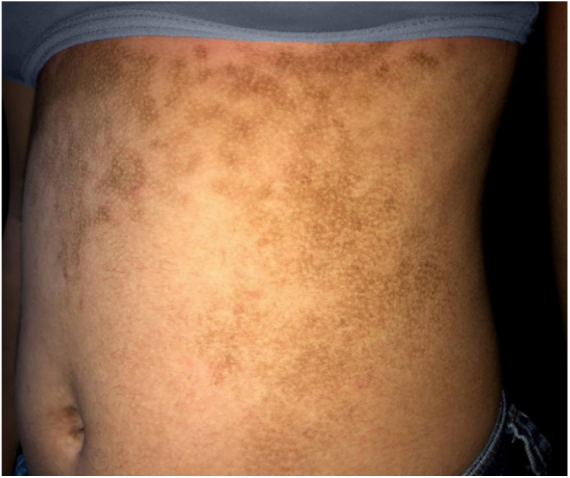
Hyperkeratotic brownish plaques with a dotted pattern affecting the abdomen.

**Figure 2:**
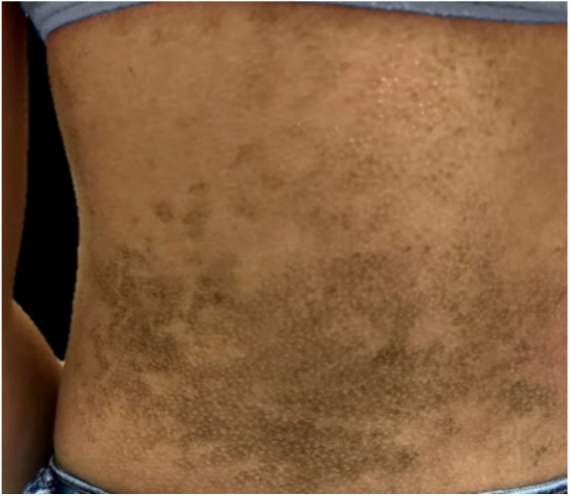
Hyperkeratotic brownish plaques with a dotted pattern affecting the back.

**Figure 3:**
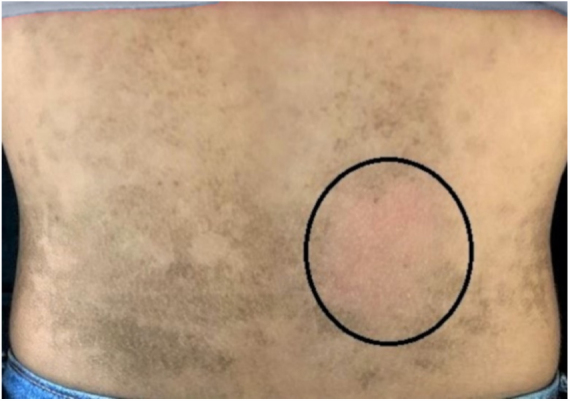
Area without lesions after rubbing the skin with gauze embedded in 70% alcohol.

“Terra firma-forme” is a Latin expression, which means “solid earth”, also known as Duncan's dirty dermatosis. It is a benign and idiopathic skin disorder that was described in 1987.^1^, ^2^, ^3^ Its prevalence and incidence are unknown, because it is an underdiagnosed disorder.^1^ The cases described in the literature have indicated greater involvement during childhood and adolescence.^2^, ^3^

The pathogenesis is uncertain; however, it is attributed to disturbance in the maturation of keratinocytes, leading to the compaction of these cells, associated with melanin, sebum, and microorganisms in the epidermis.^1^, ^2^, ^3^ this fact explains the hyperkeratosis and hyperpigmentation clinically observed.

This dermatosis is characterized by slightly elevated, brownish or blackened, hyperkeratotic, and asymptomatic plaques and papules. The lesions are typically located in the neck, face, and trunk, bilaterally or unilaterally. It affects patients with adequate hygiene habits, and the lesions are not removed by washing with soap and water.^1^, ^2^

When the presence of the condition is suspected, dermatoscopy helps by detecting brownish polygonal plates arranged in a mosaic pattern.^2^, ^3^ The diagnosis is confirmed by removing the affected area by firmly and persistently rubbing the skin with cotton soaked in 70% isopropyl alcohol.^1^, ^2^, ^3^, ^4^ This procedure possibly determines protein denaturation, interfering in the cell metabolism and diluting lipoprotein membranes,^5^ thus avoiding blood tests and skin biopsies.^4^ Histological evaluation is unnecessary; however, if performed, it can indicate acanthosis, papillomatosis, and lamellar hyperkeratosis.^3^

Differential diagnoses are made with hyperpigmented lesions, including confluent and reticulated papillomatosis of Gougerot and Carteaud, which has some similar characteristics and is considered a superficial variant of that dermatosis. It is possible to differentiate them by removing the lesions with alcohol in terra firma-forme dermatosis, in addition to considering other clinical findings, such as for example, acanthosis nigricans, which is a skin disorder usually associated with metabolic disorders and dermatitis neglecta, easily washed with soap and water,^4^ among others.^1^, ^2^

The treatment consists of the application of 70% isopropyl alcohol; however, the lesions may recur.^1^, ^2^, ^3^, ^4^ The skin should be washed with soap and water after using alcohol, taking into account the possible symptoms of intoxication as drowsiness, lethargy, respiratory depression, mucosal irritation, and others, especially in children. Absorption through the intact skin surface is low; however, it may increase after prolonged exposure.^5^ There is a report of the use of 5% salicylic acid once a day with a good response after two weeks.^3^

The patients and families should be advised to clean the skin at home,^1^ in addition to the application of emollients for preventing xerosis caused by recurrent use of alcohol.^2^

It is important to recognize this dermatosis in order to avoid incompatible diagnostic and therapeutic procedures, as well as to reassure adolescents and family members, thus reducing social impact on patients’ lives.

## Financial support

None declared.

## Authors’ contributions

Bruna Anjos Badaró: Conception and planning of the study; elaboration and writing of the manuscript; obtaining, analysis, and interpretation of the data; intellectual participation in the propaedeutic and/or therapeutic conduct of the studied cases; critical review of the literature; critical review of the manuscript.

Lucia Martins Diniz: Approval of the final version of the manuscript; conception and planning of the study; effective participation in research orientation; intellectual participation in the propaedeutic and/or therapeutic conduct of the studied cases; critical review of the literature; critical review of the manuscript.

Paulo Sergio Emerich Nogueira: Intellectual participation in the propaedeutic and/or therapeutic conduct of the studied cases; critical review of the literature.

## Conflicts of interest

None declared.
